# Cationic Amphipathic Antimicrobial Peptides Perturb the Inner Membrane of Germinated Spores Thus Inhibiting Their Outgrowth

**DOI:** 10.3389/fmicb.2018.02277

**Published:** 2018-09-26

**Authors:** Soraya Omardien, Jan Wouter Drijfhout, Sebastian A. Zaat, Stanley Brul

**Affiliations:** ^1^Swammerdam Institute for Life Sciences, Department of Molecular Biology and Microbial Food Safety, University of Amsterdam, Amsterdam, Netherlands; ^2^Leiden University Medical Centre (LUMC), Leiden University, Leiden, Netherlands; ^3^Department of Medical Microbiology, Centre for Infection and Immunity Amsterdam (CINIMA), Academic Medical Centre, University of Amsterdam, Amsterdam, Netherlands

**Keywords:** antimicrobial peptides, thrombocidin, bactericidal permeability increasing protein, lantibiotics, *Bacillus subtilis* spores, inner membrane damage

## Abstract

The mode of action of four cationic amphipathic antimicrobial peptides (AMPs) was evaluated against the non-pathogenic, Gram-positive, spore-forming bacterium, *Bacillus subtilis*. The AMPs were TC19, TC84, BP2, and the lantibiotic Nisin A. TC19 and TC84 were derived from the human thrombocidin-1. Bactericidal peptide 2 (BP2) was derived from the human bactericidal permeability increasing protein (BPI). We employed structured illumination microscopy (SIM), fluorescence microscopy, Alexa 488-labeled TC84, *B. subtilis* mutants producing proteins fused to the green fluorescent protein (GFP) and single-cell live imaging to determine the effects of the peptides against spores. TC19, TC84, BP2, and Nisin A showed to be bactericidal against germinated spores by perturbing the inner membrane, thus preventing outgrowth to vegetative cells. Single cell live imaging showed that the AMPs do not affect the germination process, but the burst time and subsequent generation time of vegetative cells. Alexa 488-labeled TC84 suggested that the TC84 might be binding to the dormant spore-coat. Therefore, dormant spores were also pre-coated with the AMPs and cultured on AMP-free culture medium during single-cell live imaging. Pre-coating of the spores with TC19, TC84, and BP2 had no effect on the germination process, and variably affected the burst time and generation time. However, the percentage of spores that burst and grew out into vegetative cells was drastically lower when pre-coated with Nisin A, suggesting a novel application potential of this lantibiotic peptide against spores. Our findings contribute to the understanding of AMPs and show the potential of AMPs as eventual therapeutic agents against spore-forming bacteria.

## Introduction

The threat of resistance development against commercially available antibiotics has spurred the search for alternative anti-infection agents, such as antimicrobial peptides (AMPs). Interest in AMPs arises from the immune modulating, anti-microbial, anti-biofilm, anti-parasite, anti-viral, and anti-cancer activities that host defence peptides have (Hoskin and Ramamoorthy, 2008; Lakshmaiah Narayana and Chen, 2015; Omardien et al., 2016; Chung and Khanum, 2017; de la Fuente-Núñez et al., 2017). AMPs are known to preferably interact with the cytoplasmic membrane of the bacterial cell, where they can permeabilize or perturb the membrane, and/or translocate to the cytosol thus possibly interacting with various cellular macromolecular components. This rather generic antibacterial activity of AMPs further increases the interest to develop them as an alternative to conventional antibiotic compounds that generally have a specific target (Marr et al., 2006). However, therapeutic use of AMPs is limited by the high production costs involved in synthesizing peptides, loss of activity due to protease degradation, and mammalian cell toxicity (Zhang and Falla, 2006). To circumvent these drawbacks of AMPs, an approach has been taken to design peptides that are shorter, less toxic, and more stable (Zhang and Falla, 2006). To achieve this goal, an in-depth understanding of the mode of action of AMPs is required for further peptide development with therapeutic potential. Knowledge acquisition concerning the mode of action of AMPs is generally based on *in vitro* studies with lipid vesicles and *in vivo* studies with living bacterial cells (Bonelli et al., 2006; Arnusch et al., 2007; Arouri et al., 2009; Sass et al., 2010; Wenzel et al., 2014; Finger et al., 2015; Khatib et al., 2016; Mitchell et al., 2016; Müller et al., 2016; Scheinpflug et al., 2017). Limited knowledge is available about the mode of action of cationic amphipathic AMPs against bacterial spores.

Spore-forming bacteria of the *Bacillales* and *Clostridiales* order produce resilient dormant spores in response to stressful environmental conditions, such as nutrient deprivation, that can withstand numerous stress conditions that would otherwise eradicate vegetative cells (Setlow, 2014b). *Bacillales* and *Clostridiales* also contain many toxin producing bacteria, such as the food-borne pathogens, *Bacillus cereus* or *Clostridium perfringens* (Wells-Bennik et al., 2016), and the health-care-associated pathogen, *Clostridium difficile* (Centers for Disease Control [CDC], 2013). Therefore, it is necessary to prevent the germination of dormant spores or the outgrowth of germinated spores into toxin producing vegetative cells. Currently, knowledge concerning the mode of action of AMPs against spores is only available for the lantibiotic subtilin produced by *Bacillus subtilis* (Liu and Hansen, 1993), the lantibiotic nisin produced by *Lactococcus lactis* (Gut et al., 2008, 2011), and the cyclic peptide bacteriocin AS-48 produced by *Enterococcus faecalis* S-48 (Abriouel et al., 2002). These peptides are active against germinated spores, i.e., the state in which the spore inner membrane is exposed. Recently, we investigated the membrane perturbation activity of three cationic amphipathic AMPs, TC19, TC84, and BP2, against *B. subtilis* vegetative cells (Omardien et al., 2018a,b). TC19 and TC84 were derived from the human platelet AMPs, thrombocidins (Zaat et al., 2015) and BP2 was designed based on the LPS-binding domains of the human bactericidal permeability increasing protein (BPI) (Abraham et al., 2003). In this study, we aimed to extend the knowledge gained on TC19, TC84, and BP2 by evaluating their effect against *B. subtilis* spores using physiological studies and live imaging. *B. subtilis* is a non-pathogenic spore-forming bacterium often used as a model organism for the pathogenic toxin producing spore formers (Stragier and Losick, 1996; Piggot and Hilbert, 2004; Setlow et al., 2017). We also included nisin (Nisin A) in our study to contribute to the available knowledge about its activity against spores and to serve as a reference peptide. Nisin is known to bind to lipid II to form defined pores and is able to interfere with spore outgrowth (Wiedemann et al., 2004; Gut et al., 2008, 2011).

## Materials and Methods

### AMP Information, Strains Used, and the Culturing Conditions

TC19, TC84, BP2, and Nisin A (>95% purity; Handary, Belgium) were dissolved in 0.01% acetic acid to reach a final concentration of 1.2 mM. The peptides were stored at -20°C and thawed on ice prior to experimentation. Strains used in the study were *B. subtilis* strain 168 from the Bacillus Genetic Stock^1^ Center, *B. subtilis* strain bSS421 (PrpsD-sfGFP) (Jahn et al., 2015), *B. subtilis* strain YK405 [MreB-green fluorescent protein (GFP)] (Kawai et al., 2009), and *B. subtilis* strain BS23 (AtpA-GFP) (Johnson et al., 2004). *B. subtilis* PrpsD-GFP produces GFP from a recombinant construct in which the GFP encoding gene is expressed under the control of the promoter for the ribosomal protein S4 gene, *B. subtilis* MreB-GFP produces the GFP fused to MreB, and *B. subtilis* AtpA-GFP produces the GFP fused to AtpA.

*Bacillus subtilis* was cultured in complete minimal medium (CMM), which consists of Spizizen’s minimal medium (SMM) (Anagnostopoulos and Spizizen, 1961), with the modifications described in Halbedel et al. (2014). The culture media of *B. subtilis* MreB-GFP and *B. subtilis* AtpA-GFP were supplemented with 0.1% xylose. For the preparation of *B. subtilis* spores, the method described by Abhyankar et al. (2011) was followed. In short, a single colony of *B. subtilis* from tryptic soy broth (TSB) solid medium was inoculated into 5 ml of TSB liquid medium. The culture was incubated overnight at 37°C while shaking at 200 rpm. The culture was subsequently re-inoculated into fresh 5 ml TSB and cultured until an optical density at an absorbance of 600 nm (OD_600_) of 0.3–0.4 was reached. A serial dilution of the culture was performed in defined minimal medium that was buffered with MOPS to pH 7.4 and supplemented with 10 mM glucose and 10 mM NH_4_Cl and incubated overnight. The culture with an OD_600_ of 0.3–0.4 was re-inoculated into defined minimal medium and cultured for 96 h until about 99% spores per ml was achieved. *B. subtilis* spores were always heat activated for 30 min at 70 °C prior to treatments and germinated in CMM containing AGFK (10 mM L-asparagine, 10 mM glucose, 1 mM fructose, and 1 mM potassium chloride).

### The Minimal Inhibitory Concentration (MIC) and Minimal Bactericidal Concentration (MBC) Against *Bacillus subtilis* Spores

To obtain the lowest concentration necessary to have an inhibitory effect on *B. subtilis* spores, the MIC was determined. To establish the lethal concentration of the AMPs against the spores, the MBC was determined. The MIC determination was performed by measuring the optical density at an absorbance of 600 nm (OD_600_) for 24 h in a microtiter plate reader (Multiskan FC, Thermo Scientific). Spores at an OD_600_ of 0.02 [1 × 10^7^ colony-forming units (CFU)/ml] were treated in CMM supplemented with AGFK. Twofold serial dilutions from 56 to 0.11 μM of the AMPs were prepared. The control consisted of CMM without AMP. The experimental conditions to determine the MBC were similar to those for the MIC, but after 24 h, the culture in every well was plated onto LB solid medium. The MIC was considered to be the lowest concentration with an increase in OD_600_ of not more than 20% of the initial OD_600_. The MBC was defined as the concentration where 99.9% of the culture produced no CFU after 24 h. Three biological and two experimental repeats were performed. The Student’s *t*-test was applied, where the difference in mean between the two groups were considered greater than would be expected by chance if the *p*-value is ≤0.05.

### Structure Illumination Microscopy (SIM) Imaging of the Membrane After Peptide Treatments

*Bacillus subtilis* PrpsD-sfGFP constitutively produce the GFP under control of the promoter for ribosomal protein S4. *B. subtilis* PrpsD-GFP spores were used to observe membrane perturbation and cytosol leakage. The spores were incubated in CMM with AGFK at an OD_600_ of 0.2 [1 × 10^8^ colony-forming units (CFU)/ml] and treated with 12 μM TC19, 12 μM TC84, 7 μM BP2, and 4 μM Nisin A for 30 min. Following treatment, the spores were stained with Nile red (0.5 μg/ml final concentration) for 5 min to visualize the membrane.

*Bacillus subtilis* MreB-GFP and *B. subtilis* AtpA-GFP spores were used to confirm the location of the inner membrane and to confirm the presence of the cell-shape determinant protein, MreB, and AtpA synthase, AtpA, in dormant and germinated spores. The inner membrane of *B. subtilis* MreB-GFP and *B. subtilis* AtpA-GFP spores were stained by adding 2 μg/ml FM 4-64 to the culture in its exponential growth phase during the sporulation process (Cowan et al., 2004). The final dormant spores will contain an FM 4-64 stained inner membrane. During germination, the FM 4-64 signal is lost, therefore the germinated spores were re-stained with FM 4-64. Dormant spores were also re-stained with FM 4-64 as a control.

The Nikon N-SIM E microscope was used for visualizing the spores. The coverslips were coated with L-dopamine to reduce binding of Nile red or FM 4-64 to avoid distortion of the structured illumination pattern projections (Zhang et al., 2013; Strahl et al., 2015). The Nikon Ti N-SIM was equipped with a CFI SR Apochromat TIRF 100× oil objective (NA1.49), a LU-N3-SIM laser unit, an Orca-Flash 4.0 sCMOS camera (Hamamatsu Photonics K.K.), and NIS-elements Ar software. Microscopy images were analyzed in ImageJ^2^

### Determining the Location of Peptide TC84 Using Alexa-488 Labelling

TC84 was N-terminally labeled with the green fluorescent dye Alexa Fluor 488 (Omardien et al., 2018b) to determine the location of the peptide after treatment. *B. subtilis* 168 spores were incubated in CMM with AGFK at an OD_600_ of 0.2 and treated with 12 μM Alexa488-TC84 for 30 min. Combination treatments of Alexa 488-TC84 and TC84 at a 1:1 w/w mixture, to have a final concentration 12 μM, were also performed. Following treatment, spores were washed thrice with PBS to remove residual labeled peptide. SIM images of spores treated with Alexa 488-TC84 combined with TC84 were also performed. Treated cells were stained with Nile red prior to SIM imaging. Imaging was performed with a Nikon Ti N-SIM and Olympus BX-60. The Olympus BX-60 was mounted with a CoolSnap fx (Photometrics) CCD camera and an UPLANFl 100×/1.3 oil objective (Tokyo, Japan). Microscopy images were analyzed in ImageJ (see text footnote 2).

### Live Imaging of *B. subtilis* Spore Outgrowth

Heat activated spores were observed over time when treated with the AMPs in three different test conditions: (1) AMPs were added to the culturing medium at lethal concentrations, (2) AMPs were added at sub-lethal concentrations to the culturing medium, and (3) spores were coated with lethal concentrations of AMPs for 5 min, followed by washing of the spores in sterile MilliQ water prior to culturing on AMP-free medium. The culturing medium contained CMM, AGFK, and 1% agarose. The time scale of 4.5 h was selected as the untreated *B. subtilis* spores overgrew the imaging point at this time in our test conditions. Lethal concentrations of the AMPs were considered to be concentrations that prevented outgrowth for 4.5 h. Sub-lethal concentrations were considered the highest concentrations of peptide that permitted spore outgrowth within 4.5 h. Microscope slide preparation and imaging were performed as described by Pandey et al. (2013). Live imaging microscopy was performed with the Nikon Eclipse Ti equipped with a Prior Brightfield LED for phase-contrast microscopy and Lumencor Spectra X LED Light Engine for fluorescence microscopy, a Nikon CFI Plan Apo Lambda 100× Oil, C11440-22CU Hamamatsu ORCA flash 4.0 camera, LAMBDA 10-B Smart Shutter from Sutter Instrument, an OkoLab stage incubator, and with NIS elements software version 4.50.00. The start of germination, germination time, burst time, and generation time of each spore were observed and measured using the ImageJ plugin SporeTrackerX designed by Norbert Vischer (Omardien et al., 2018c). Statistical analysis was performed in SigmaPlot 13.0. The start of germination was considered to be the time point when phase bright spores turn phase dark, and the germination time was considered the time of the phase-bright to phase-dark transition (Pandey et al., 2013, 2015). The start of germination and end of germination are determined with SporeTrackerX by measuring the change in intensity of the center region of the marked spore and comparing it to a pre-defined threshold setting. The germination time is the difference in time between the start and end of germination. The burst time is the time when the spore-coat is shed, which is automatically noted by the macro when a “jump-like” increase in area during outgrowth occurs or can be manually added. The generation time was determined by the macro by finding the contour of the growing cell or colony in each frame and measuring the area over time (Pandey et al., 2013, 2015).

## Results

### The AMPs Are Bactericidal Against *B. subtilis* in Standard Growth and Survival Tests Conditions

The MIC and MBC were determined to obtain the lowest peptide concentration necessary to evaluate the mode of action of the AMPs against *B. subtilis* strain 168 spores. The MIC and MBC values for spores were for each of the individual AMPs not significantly different (*p*-value >0.05, **Table 1**). This shows that there is no discernible window that allow spore-forming bacteria to adapt phenotypically to the presence of the AMPs. In the subsequent experiments, we evaluate the mode of action of the AMPs against *B. subtilis* spores to discern whether spore germination or outgrowth is affected. The concentrations selected were 12 μM TC19, 12 μM TC84, 7 μM BP2, and 4 μM Nisin A.

**Table 1:** Minimal inhibitory concentration (MIC) and minimal bactericidal concentration (MBC) of the antimicrobial peptides against spores of *Bacillus subtilis* strain 168 in standard microbial growth and survival tests conditions.

Antimicrobial peptide	MIC (μM)	MBC (μM)
	
	Mean ± SD	Mean ± SD
TC19	9.3 ± 3.2	5.8 ± 1.4
TC84	5.8 ± 1.4	4.7 ± 1.5
BP2	3.5 ± 1.1	3.5 ± 1.1
Nisin A	1.0 ± 1.1	2.0 ± 2.0


*An estimate for Alexa488-labeled TC84 is discussed in the text.*

### TC19, TC84, BP2, and Nisin a Target the Inner Membrane of Germinated Spores

**FIGURE 1:**
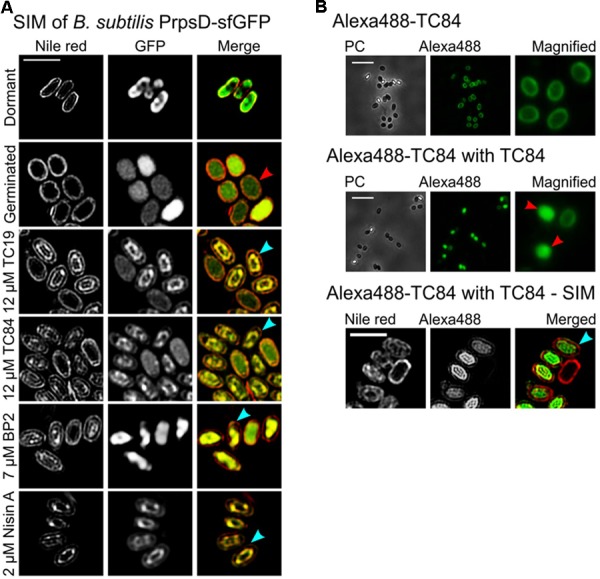
The effects of TC19, TC84, BP2, and Nisin A on *Bacillus subtilis* spores. **(A)** SIM imaging of treated and untreated Nile red stained *B. subtilis* PrpsD-sfGFP spores. Nile red stained the spore coat of untreated dormant spores. The spore-coat, spore outer membrane, and spore inner membrane of germinated spores was also stained with Nile red. Treatment with the AMPs showed that the peptides target germinated spores causing the inner membrane to appear irregular or shrunken (turquoise arrows) compared to the untreated germinated spores (red arrow). **(B)** *B. subtilis* 168 treated with Alexa488-TC84, with and without TC84. Treatment with only Alexa488-TC84, followed by multiple washing steps to remove residual AMP, showed that the labeled peptide binds to the spore coat of both dormant and germinated spores. Combining Alexa488-TC84 with TC84 showed Alexa488-TC84 present in the spore core (red arrows), suggesting inner membrane perturbation. Nile red stained *B. subtilis* 168 spores treated with Alexa488-TC84, combined with TC84, also showed an irregular and shrunken inner membrane (turquois arrow). Treatment was for 30 min with 12 μM TC19, 12 μM TC84, 7 μM BP2, and 4 μM Nisin A, respectively. Treatments were with only 12 μM Alexa488-TC84 or with a combination of 6 μM Alexa488-TC84 and 6 μM TC84. Scale bar represent 2 μm.

TC19, TC84, BP2, and Nisin A were only active against *B. subtilis* spores once germination, i.e., uptake of water and release of calcium dipicolinic acid, had occurred. Structure illumination microscopy (SIM) images of Nile red stained *B. subtilis* PrpsD-sfGFP spores made it possible to visualize the inner membrane of germinated spores (**Figure 1A**). Nile red stained dormant spores suggesting that the dye binds to the spore coat or outer membrane. PrpsD-sfGFP protein was located in the spore coat and core, the former presumably due to the mother cell expression of the PrpsD-sfGFP ribosomal protein. Germinated untreated PrpsD-sfGFP spores appeared swollen compared to the dormant spores, with Nile red staining the spore-coat or outer membrane and the inner membrane (**Figure 1A**, red arrow). After treatment with the AMPs, the Nile red stained spore coat or outer membrane remained intact, but the inner membrane appeared shrunken and distorted (**Figure 1A**, turquoise arrows) suggesting that the spore inner membrane was perturbed. Unlike after treatment of vegetative cells where leakage of PrpsD-sfGFP occurred (Omardien et al., 2018b), this fusion protein remained within the core of the germinated spores and no visible core leakage was observed. Leakage of PrpsD-sfGFP might have been hindered by the outer spore coat.

To confirm membrane perturbation, Alexa488-labeled TC84 was employed. Alexa488-labeled TC84 was used as a proxy for TC84 and observation made with Alexa488-labeled TC84 should be interpreted with caution. Alexa488-labeled TC84 did not have a comparable antimicrobial effect to that of TC84 against *B. subtilis* vegetative cells, when comparing the MIC (Omardien et al., 2018b). Only the physiological effect, by creating fluid domains in the membrane, were similar between Alexa488-labeled TC84 and TC84. A concentration of 7 μM TC84 prevented the growth of 1 × 10^7^ vegetative cells, whereas 56 μM Alexa488-labeled TC84 was unable to completely prevent the outgrowth of 1 × 10^7^ or 1 × 10^5^ cells after about 13 h of treatment. Since the MBC values for TC84 against vegetative cells and spores were not significantly different (Omardien et al., 2018a and **Table 1** of the current paper), we estimate that the effect of Alexa488-labeled TC84 will be similar against vegetative cells and spores. In this study, the Alexa488-labeled TC84 should be viewed as a fluorescent probe that behaves similar but certainly not identical to the native TC84 peptide.

At a low concentration, Alexa488-TC84 bound to the spore-coat and/or inner membrane of spores that had germinated. Alexa488-TC84 was unable to permeabilize the inner membrane of the spore, but when combined with unlabeled TC84, it accumulated within the germinated spore core (**Figure 1B**, red arrows). SIM and Nile red staining were employed to confirm the observation with Alexa488-TC84 when combined with TC84 (**Figure 1B**). The SIM images showed that Nile red stained the spore coat and inner membrane of the germinated spore. Alexa488-TC84 was observed on the surface of the spore coat and accumulated in the spore core (**Figure 1B**, turquoise arrow). This finding suggested that the inner membrane of germinated spores is perturbed by TC84. A similar phenomenon might be occurring during the treatment of spores with the membrane active TC19, BP2, and Nisin A as these peptides have shown to permealize the vegetative cell membrane (Breukink et al., 1999; Omardien et al., 2018b).

**FIGURE 2:**
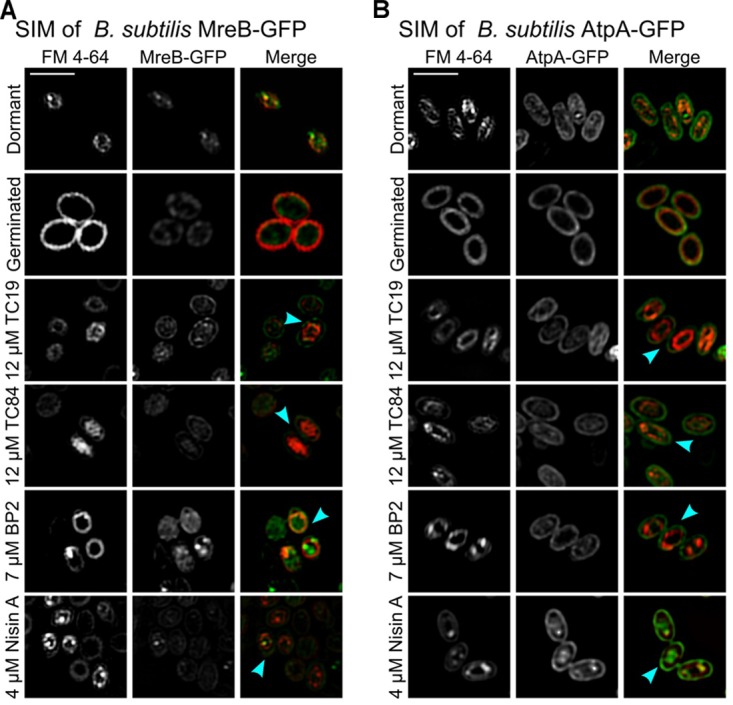
SIM imaging of FM 4-64 stained spores. FM 4-64 stained the inner membrane of both **(A)** *B. subtilis* strain MreB-GFP and **(B)** *B. subtilis* strain AtpA-GFP dormant and germinated spores. The presence of protein MreB-GFP and AtpA-GFP could be observed in both dormant and germinated spores. However, the MreB-GFP and AtpA-GFP were also located outside the inner membrane, presumably originating from the mother cell membrane that forms the outer membrane of the spore. After treatment with the AMPs, all the treated germinated spores had an irregular or shrunken (turquoise arrow) inner membrane compared to the untreated germinated spores. Treatment was for 30 min with 12 μM TC19 and TC84, 7 μM BP2, and 4 μM Nisin A. Scale bar represent 2 μm.

The peripheral membrane protein, MreB, delocalizes from the membrane when the membrane potential is lost after treatment with proton ionophore CCCP (Strahl and Hamoen, 2010), lipopeptide daptomycin (Müller et al., 2016), and synthetic cyclic hexapeptide cWFW (Scheinpflug et al., 2017). Previously, we showed that TC19, TC84, and BP2 cause delocalization of essential membrane bound proteins, including MreB, due to the distortion of the membrane by the peptides (Omardien et al., 2018a). MreB is an important protein for lateral cell elongation during cell growth (Carballido-López and Formstone, 2007). Using *B. subtilis* MreB-GFP, we were able to establish the presence of MreB in dormant spores, and that the protein remains present after germination and at the onset of outgrowth (**Figure 2A**). However, MreB-GFP was not unequivocally patch-wise localized in the germinated spores as it is in vegetative cells (Strahl et al., 2014). Peptide-induced delocalization of MreB-GFP was not observed (**Figure 2A**, turquoise arrows). *De novo* MreB synthesis was shown to occur at 60 min after the onset of germination in the outgrowth phase (Sinai et al., 2015) at which point patch-wise localization as observed in vegetative cells will occur. The integral membrane-bound protein, AtpA, crucial to ATP metabolism in the cell, was synthesized at 30 min after the onset of germination (Sinai et al., 2015). TC19, TC84, and BP2 have been shown to alter the localization of AtpA in vegetative cells (Omardien et al., 2018a). Using *B. subtilis* AtpA-GFP, the presence of AtpA-GFP in dormant and germinated spores were confirmed (**Figure 2B**). AtpA-GFP was localized at the outside and inside of the inner membrane. Treatment with TC19, TC84, and BP2 did not notably alter the AtpA-GFP location at the germinated spore membrane as with vegetative cells (**Figure 2B**, turquoise arrows).

Unlike Nile red, FM 4-64 did not bind to the spore coat. We stained the inner membrane of *B. subtilis* MreB-GFP and *B. subtilis* AtpA-GFP spores with FM 4-64 during the sporulation process to independently visualize the spore inner membrane (Cowan et al., 2004). Re-staining the dormant spores with FM 4-64 were used to confirm that only the outer and inner membrane of *B. subtilis* MreB-GFP was stained and that the spore coat remained unstained. FM 4-64 staining also confirmed the swelling of the inner membrane during germination. Treatment of *B. subtilis* MreB-GFP and *B. subtilis* AtpA-GFP with the AMPs confirmed the shrinking of the inner membrane (**Figures 2A,B**, turquoise arrows). MreB-GFP and AtpA-GFP were present outside the inner membrane, as for instance, observed in spores treated with TC19 (**Figures 2A,B**). The presence of MreB-GFP and AtpA-GFP outside the inner membrane presumably originates from the mother cell membrane that forms the outer membrane of the spore. Perturbation of the inner membrane will obstruct the normal functioning of essential membrane bound proteins such as MreB and AtpA, thus perturbing cell elongation or metabolism that will most likely prevent spore outgrowth.

### Live Imaging of Spores in the Presence of TC19, TC84, and BP2

**FIGURE 3:**
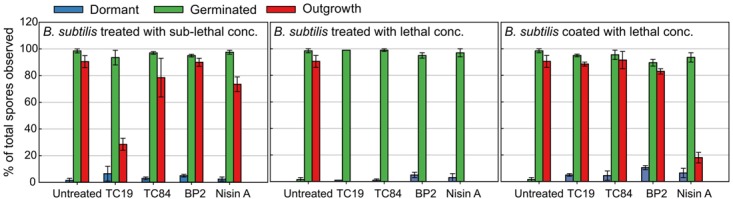
Quantification of live imaging data. The number of spores that remained dormant, germinated, or grew out into vegetative cells (outgrowth) after treatment for 4.5 h were quantified. Spores were coated and exposed to lethal concentration of the peptides (32 μM TC19, 23 μM TC84, 56 μM BP2, and 7 μM Nisin A) in the culturing medium. Sub-lethal concentrations of the peptides (32 μM TC19, 23 μM TC84, 56 μM BP2, and 7 μM Nisin A) were also added to the culturing medium. Standard error bars represent the data of two biological repeats.

Live imaging was employed to evaluate whether the peptides influence the start of germination, germination duration, the shedding of the spore coat (burst time), and the generation time of the outgrowing spores (vegetative cells) after treatment. Differences in efficacy of the AMPs against *B. subtilis* spores could be observed between liquid culturing medium and solid culturing medium. Therefore, the concentrations used during the live imaging analysis were changed to lethal concentrations (32 μM TC19, 23 μM TC84, 56 μM BP2, and 7 μM Nisin A) and sub-lethal concentrations (16 μM TC19, 12 μM TC84, 28 μM BP2, and 3.5 μM Nisin A). Where lethal concentrations were considered, the highest concentration of AMP required to prevent spore outgrowth, and sub-lethal concentrations were the highest concentration of AMP required to enable spore outgrowth during the live imaging of 4.5 h. The percentage of spores was quantified after 4.5 h of imaging to obtain an overview of the outcome of the different AMP treatments (**Figure 3**). Of the total number of spores observed, about 98% germinated and 2% remained dormant when the spores were untreated. Of the spores that germinated, 92% grew out into vegetative cells. The Student’s *t*-test was employed and the results showed that at sub-lethal concentrations the number of spores that remained dormant, germinated, or grew out were not statistically different from the untreated spores. Only the sub-lethal treatment with TC19 caused less spores to grow out into vegetative cells, most likely due to the concentration of TC19 used. At lethal concentrations, there was no statistical significance between the percentage of spores that were dormant or germinated compared to the untreated spores. As expected, the lethal concentrations prevented the outgrowth of spores into vegetative cells for the duration of 4.5 h of live imaging.

The data obtained after analyzing the live images with SporeTrackerX failed the Shapiro–Wilk normality test; therefore, the alternative Mann–Whitney test was used to assess possible differences between treatments (McDonald, 2014). The Mann–Whitney test considers the difference in median values between the two groups, where the difference is greater than would be expected by chance if the *p*-value is smaller than 0.05. The live imaging results are summarized in **Table 2** where the median, *p*-value, inter-quartile range (IQR), and counts can be found. Additional data obtained after analyzing the live images with SporeTrackerX can be found in the **Supplementary Material**.

**Table 2:** Live imaging results after treatment of heat activated *Bacillus subtilis* strains 168 dormant spores with the peptides.

Treatment		Start of germination (min)				Germination time (min)				Burst (min)				Generation time (min)			
				
		Median	*p*-value	IQR	Counts	Median	*p*-value	IQR	Counts	Median	*p*-value	IQR	Counts	Median	*p*-value	IQR	Counts
Untreated		17		10	384	4		1	384	120		25	369	44		9	345

TC19	Sub-lethal	16	0.34	13	174	4	0.03	2	174	142	≤0.01	38	52	44	0.59	5	49
	Lethal	18	0.08	10	370	4	≤0.01	2	370								
	Coated	14	0.03	12	228	4	0.01	1	228	110	≤0.01	25	168	42	≤0.01	3	151
TC84	Sub-lethal	17	0.07	10	212	4	≤0.01	1	212	105	≤0.01	25	117	41	≤0.01	4	129
	Lethal	16	0.98	12	342	4	≤0.01	2	342								
	Coated	18	≤0.01	15	209	4	≤0.01	1	209	107	≤0.01	39	188	40	≤0.01	3	177
BP2	Sub-lethal	15	0.17	11	380	4	≤0.01	1	380	115	0.03	27	317	53	≤0.01	33	288
	Lethal	18	0.02	13	499	4	≤0.01	2	499								
	Coated	16	0.89	13	325	4	≤0.01	1	325	110	≤0.01	28	270	43	0.47	7	210
Nisin A	Sub-lethal	16	0.7	13	169	4	≤0.01	1	169	114	0.19	28	52	39	≤0.01	3	52
	Lethal	16	0.26	12	354	3	≤0.01	1	354								
	Coated	15	0.68	12	169	4	≤0.01	1	169	113	0.43	48	33	40	≤0.01	3	28


*Coated and lethal concentrations were 32 μM TC19, 23 μM TC84, 56 μM BP2, and 7 μM Nisin A.*

*Sub-lethal concentrations were 16 μM TC19, 12 μM TC84, 28 μM BP2, and 3.5 μM Nisin A.*

*IQR refers to inter-quartile range.*

**FIGURE 4:**
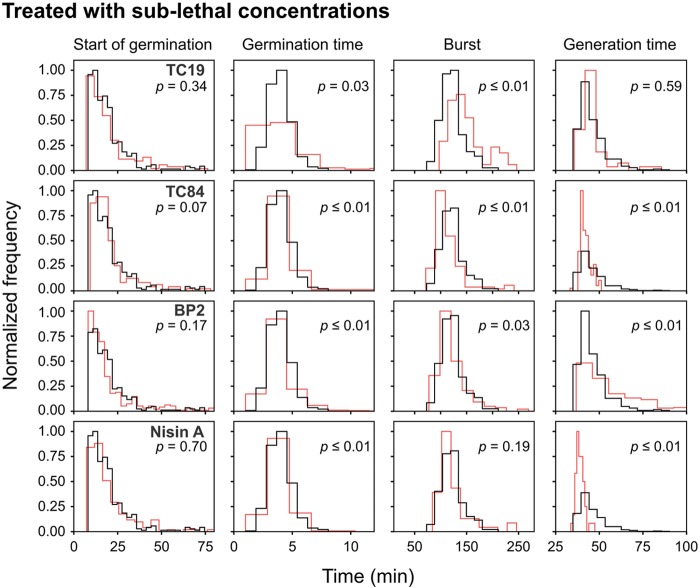
Frequency distribution curves of *B. subtilis* spores treated with sub-lethal peptide concentrations. Sub-lethal peptide concentrations were 16 μM TC19, 12 μM TC84, 28 μM BP2, and 3.5 μM Nisin A. Treated conditions (red line) were overlaid with untreated conditions (black line). The histogram was normalized to occupy an area of one and was rescaled so that the maximum value in the histogram is equal to one. Significance were determined using the Mann–Whitney test, where a statistically significant difference with a *p*-value ≤0.05 is obtained if the median values between the two groups is greater than would be expected by chance. Observations of two biological repeats were grouped and analyzed as one data set.

**FIGURE 5:**
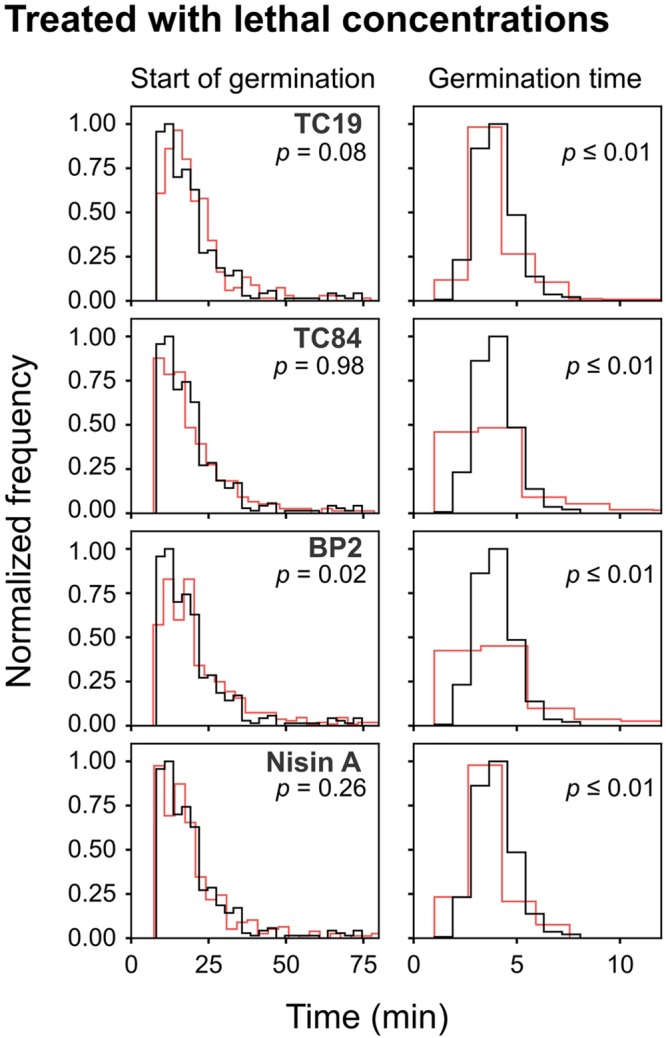
Frequency distribution curves of *B. subtilis* spores treated with lethal peptide concentrations. Lethal peptide concentrations were 32 μM TC19, 23 μM TC84, 56 μM BP2, and 7 μM Nisin A. Treated conditions (red line) were overlaid with untreated conditions (black line). The histogram was normalized to occupy an area of one and was rescaled so that the maximum value in the histogram is equal to one. Significance was determined using the Mann–Whitney test, where a statistically significant difference, with a *p*-value ≤0.05, is obtained if the median values between the two groups are greater than would be expected by chance. Observations of two biological repeats were grouped and analyzed as one data set.

To observe the effects of the AMPs on the germination heterogeneity, the germination process after treatment at sub-lethal and lethal concentrations were evaluated. Treatment of the spores with sub-lethal concentrations of the AMPs seemed to delay the start of germination, but the statistical analyses did not show a significant difference (**Table 2** and **Figure 4**). Treatment with lethal concentrations of TC19, TC84, and Nisin A did not significantly affect the start of germination, but BP2 significantly delayed (started later) the start of germination (**Table 2** and **Figure 5**). Even though a significant difference was obtained for the start of germination when treated with lethal concentrations of BP2, the difference was about 1 min and therefore considered negligible. The germination time of the spores when treated with sub-lethal and lethal concentrations of the AMPs were significantly different from that of untreated spores, but the effects were about 1 min and considered negligible. Overall, the results suggest that lethal and sub-lethal concentrations of the AMPs do not affect the germination process of *B. subtilis* spores.

To evaluate the effects of the AMPs on the busting of the bacterial cell out of the spore coat, the burst time was observed. Treatment with sub-lethal concentrations of the AMPs caused a significant difference in burst time, except when spores were treated with Nisin A (**Table 2** and **Figure 4**). TC19 significantly delayed (started later) the burst time with a median of 22 min. TC84 and BP2 significantly shortened (started earlier) the burst time with a median of 15 min and 5 min, respectively. Lethal concentrations prevented the bursting of germinated spores, as was expected. The results suggest that the treatment with the AMP do have an effect on the burst time of germinated spore.

To observe the effects of the AMPs on vegetative cell growth, the generation time was observed. Sub-lethal concentrations of TC19 had no effect on the generation time, but TC84 and Nisin A significantly increased the generation time with a median of 3 and 5 min, respectively (**Table 2** and **Figure 4**). BP2 showed to significantly delay the generation time by 9 min. Lethal concentrations prevented vegetative cell growth of germinated spores. The results suggest that the treatment with the AMPs do have an effect on the generation time.

**FIGURE 6:**
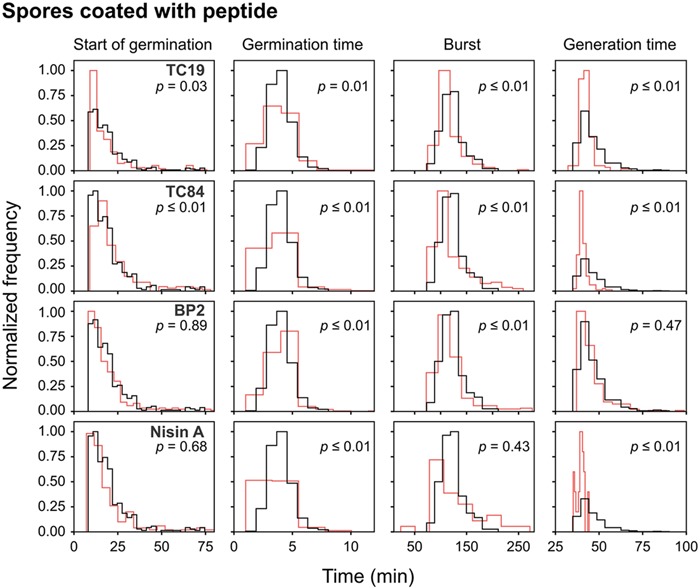
Frequency distribution curves of spores coated with the peptides. *B. subtilis* spores were pre-coated with lethal peptide concentrations, which were 32 μM TC19, 23 μM TC84, 56 μM BP2, and 7 μM Nisin A. Treated conditions (red line) were overlaid with untreated conditions (black line). The histogram was normalized to occupy an area of one and was rescaled so that the maximum value in the histogram is equal to one. Significance were determined using the Mann–Whitney test, where a statistically significant difference, with a *p*-value ≤0.05, is obtained if the median values between the two groups are greater than would be expected by chance. Observations of two biological repeats were grouped and analyzed as one data set.

Alexa488-TC84 was found to bind to the crust or outer coat of dormant and germinated spores (**Figure 1B**). Therefore, we pre-coated the dormant spores in lethal concentrations of the peptides to evaluate whether the peptides bound to the spore coat can prevent outgrowth once germination occurs. Quantification of the number of spores that germinated after coating with the peptides showed that there was no statistical significance in the percentage of dormant and germinated spores compared to the untreated spores (**Figure 3**). The live imaging results showed that coating with TC19 significantly delayed the start of germination by 3 min, but TC84 significantly shortened the time to the start of germination by 1 min (**Table 2** and **Figure 6**). The influence of BP2 and Nisin A on the timing of the start of germination was not significantly different. The germination time was significantly different but the effect was negligible as it was about 1 min. A delay in burst time was observed for TC19, TC84, and BP2 with a median of 10, 13, and 10 min, respectively. Nisin A shortened the burst time with a median of 7 min, but the effect was not significant. The generation time was significantly different, except for treatment with BP2. TC19, TC84, and Nisin A shortened the generation time with a median of 2, 4, and 4 min, respectively. Overall, these finding suggest that the coating of dormant spores with the peptides did not drastically affect the germination process of *B. subtilis* spores, but did affect the burst time and the generation time.

Interestingly, after coating with Nisin A, only 19% of the spores burst and went on to the outgrowth process (**Figure 3**), which was statistically significant with a *p*-value of 0.01. By contrast 89, 90, and 84% of the spores coated with TC19, TC84, and BP2, respectively, burst and grew out into vegetative cells, showing that these peptides had no effect. Nisin A appeared to affect the shedding of the spore-coat of germinated spores through an as yet unknown mechanism that warrants further investigation. Nisin A might be creating a layer of AMP that directly targets the spore inner membrane once germination occurs.

## Discussion

Previously, we showed that TC19, TC84, and BP2 distort the membrane of *B. subtilis* vegetative cells by creating fluid domains, which leads to membrane permeabilization and delocalization of membrane bound proteins (Omardien et al., 2018a,b). TC19, TC84, and BP2 were also rapidly bactericidal without readily inducing resistance development (Omardien et al., 2018a). We aimed in this study to evaluate the membrane perturbation effects of TC19, TC84, and BP2 together with Nisin A on *B. subtilis* spores. TC19, TC84, BP2, and Nisin A showed to be bactericidal against germinated spores, similar to what was reported for the lantibiotics subtilin and nisin, and cyclic peptide bacteriocin (Liu and Hansen, 1993; Abriouel et al., 2002; Gut et al., 2008, 2011). Generally, dormant spores consist of various layers not present in vegetative cells: an exosporium, a coat layer, an outer membrane, and a peptidoglycan (PG) rich cortex (Setlow, 2014a). The cortex covers the PG germ cell wall (GCW) and inner membrane that are similar to the vegetative cell wall and membrane. However, the dormant spore’s inner membrane has a lower lateral mobility, contains more cardiolipin, and has a higher viscosity compared to the vegetative cell membrane (Cortezzo et al., 2004; Cowan et al., 2004; Cortezzo and Setlow, 2005; Kawai et al., 2006; Setlow, 2006; Sunde et al., 2009; Loison et al., 2013). All the mentioned layers cover the inner spore core that contains various properties which contribute to the dormant spore’s resistance. The inner spores core has a low water content, high level of dipicolinic acid (DPA) together with various divalent cations, and α/β-type small acid-soluble spore proteins (SASPs) that protects the DNA against damage (Setlow, 2006, 2007, 2013, 2014b). When the dormant spore germinates, the above-mentioned properties are lost, the spore loses its resistance, and at this stage, the peptides gain access to the inner membrane.

Germination of spores leads to the release of CaDPA, cortex hydrolysis, and rehydration of the spore core causing swelling, followed by the increase in mobility of the macromolecular components and that of the inner membrane (Setlow, 2003; Setlow et al., 2017). Protein synthesis has also been reported to occur during germination, but it still remains a subject of debate (Sinai et al., 2015; Boone and Driks, 2016; Korza et al., 2016). The spore inner membrane is remodeled, expands, and become permeable (Setlow et al., 2017). The GCW is also remodeled and surrounds the inner membrane (Setlow et al., 2017). The spore coat is only released during the burst, but it remains unclear what happens to the outer spore membrane during germination. Presumably, it is lost during the cortex hydrolysis or is shed with the spore coat, but to the best of our knowledge, it is unclear what happens to the outer membrane during germination and outgrowth. With the aid of SIM, the fluorescent membrane dyes Nile red and FM 4-64, and the Alexa488-labeled TC84, we were able to establish with confidence that the spore inner membrane is indeed perturbed after AMP treatment. TC19, TC84, BP2, and Nisin A treatment caused the inner membrane of germinated spores to appear shrunken compared to the untreated spores and Alexa488-labeled TC84 was present in the spore core when combined with unlabeled TC84. Membrane invagination was observed in vegetative cells treated with TC19, TC84, and BP2 (Omardien et al., 2018b); therefore, the shrunken inner membrane might be invaginated too after peptide treatment.

Rehydration of the core facilitates protein mobility and enzyme activity. ATP, nucleotides, and amino acids are generated, and macromolecules are synthesized from endogenous resources (Paidhungat and Setlow, 2002; Setlow, 2003; Keijser et al., 2007; Sinai et al., 2015), processes vital for outgrowth. We evaluated the effects of TC19, TC84, BP2, and Nisin A at 30 min after the addition of the germinant AGFK. During this period, proteins crucial for vegetative cell metabolism are made such as ATP synthesis and glycolysis (Sinai et al., 2015). The ATP synthase protein AtpA was shown to already be present in dormant spores and further synthesized after 30 min of germination (Sinai et al., 2015). Disturbance of the activity of this protein might prevent the subsequent outgrowth phase. We have shown with vegetative cells that the location of the integral membrane bound protein, AtpA, was irregular due to the membrane perturbation effects of TC19, TC84, and BP2 (Omardien et al., 2018a). Other essential membrane bound proteins that were delocalized after TC19, TC84, and BP2 treatment were the cell wall synthesis proteins MurG, MraY, PBP2b, PonA, MreB, and FtsW, cell membrane synthesis proteins PgsA and PlsX, and cell division proteins FtsZ and DivIVA (Omardien et al., 2018a). The localization of the succinate dehydrogenase protein SdhA, involved in the Kreb’s cycle and respiration, was also affected by the membrane perturbation activity of TC19, TC84, and BP2. Protein MurG, MraY, PonA, MreB, and FtsZ have been shown to be synthesized later during ripening at 60 min during germination or during the initiation of outgrowth (Sinai et al., 2015). We observed in this study the presence of AtpA-GFP and MreB-GFP in dormant spores, and in germinated spores after 30 min of culturing. However, irregular localization of MreB-GFP or AtpA-GFP was not observed. Whether this is a true biological difference between the spore membrane and vegetative cells, or whether it is due to the limit of our resolution and the “shrunken” membrane, remains to be assessed. Based on our findings with vegetative cells, we conclude that the membrane perturbation caused by TC19, TC84, and BP2 will affect the correct localization of essential membrane bound proteins and thus prevent the initiation of normal processes required for outgrowth.

To obtain an overview of the effect of TC19, TC84, BP2, and Nisin A on the germination and outgrowth of dormant spores, single spore observations using live imaging was employed. At lethal and sub-lethal concentrations, the germination process was not considerably affected, but the burst and generation time was. These observations are similar to what was observed for tea compounds (Pandey et al., 2014), sorbic acid (Pandey et al., 2015), and synthetic compounds (Omardien et al., 2018c). However, the effects were not as extreme. Coating of the spore with lethal concentrations of TC19, TC84, BP2, and Nisin A also affected the burst and generation time of outgrowing spores. Coating of spores with TC19, TC84, and BP2 did not prevent outgrowth into vegetative cells, but the lantibiotic Nisin A prevented the outgrowth of 79% of the germinated spores. This observation suggests a possible novel application for this peptide in spore outgrowth prevention.

## Conclusion

In conclusion, we show for the first time the spore inner membrane’s perturbation by cationic amphipathic AMPs TC19, TC84, BP2, and Nisin A. TC19, TC84, and BP2 showed to be promising candidates as antimicrobial agents against the germinating spores of Gram-positives. The Nisin A data hinted at a potential additional application for lantibiotic peptides in providing a bactericidal coating for the spores that warrants further investigation. Such an investigation is currently underway with the food-borne pathogen *Bacillus cereus* and health-care associated pathogen *Clostridium difficile*.

## Author Contributions

SO conducted the research and wrote the manuscript. JD was involved in the design of the peptides. SZ was involved in the design of the peptides, supervised the study, and edited the manuscript. SB supervised the study and edited the manuscript.

## Conflict of Interest Statement

The authors declare that the research was conducted in the absence of any commercial or financial relationships that could be construed as a potential conflict of interest.

## Footnotes

1 http://www.bgsc.org/

2 https://imagej.nih.gov/ij/
